# Contribution of the World Health Organization/ the special program for research and training in tropical disease (WHO/TDR’s) in institution building: lessons from Ethiopia

**DOI:** 10.1186/s12913-023-09767-z

**Published:** 2023-07-18

**Authors:** Mirgissa Kaba, Kalkidan Solomon, Yayehyirad Kitaw, Derbew Fikadu Berhe, Alemseged Abdissa

**Affiliations:** 1 Department of Preventive Medicine, School of Public Health, College of Health Sciences, Addis Ababa University, Addis Ababa, Ethiopia; 2 A private senior research consultant, Addis Ababa, Ethiopia; 3 School of pharmacy, College of Health Sciences, Addis Ababa University, Addis Ababa, Ethiopia; 4 Center for equitable Global Surgery, University of Global Health Equity, Kigali, Rwanda; 5 Armauer Hansen Research Institute, Addis Ababa, Ethiopia

**Keywords:** WHO/TDR, AHRI, Impact, Lesson learnt, Sustainability

## Abstract

**Background:**

World Health Organization/Tropical Disease Research (WHO/TDR) has enduring investment in transfers of skills critical to sustaining resilient health research systems through postgraduate training, clinical research and development fellowship (CRDF), bioethics, and grants to neglected tropical disease research. TDR has a long history of partnership with Armauer Hansen Research Institute (AHRI) in Ethiopia. The collaboration started with individuals and lead to institution survival and success. Therefore, the purpose of this study was to explore the impact and lessons learned of TDR initiatives in Ethiopia.

**Method:**

This study was guided by the ‘TDR Impact Pathways’. A total of thirteen in-depth, and five key informant interviews were conducted with individuals who are currently working in Addis Ababa, Gondar, Jimma Universities and AHRI. In addition to the interviews, reports, written communications and publications were reviewed. Interviews were audio recorded, transcribed verbatim, inductively coded, and analyzed thematically. The results were presented following the themes with supportive verbatim quotes.

**Conclusion:**

TDR’s seed grants, training opportunities and technical support catalyzed individual, institutional and national research capacity in Ethiopia. This is a useful indication of how long-term collaboration between individuals could have broader institutional implication as evidenced from the TDR-AHRI complementary partnership.

**Supplementary Information:**

The online version contains supplementary material available at 10.1186/s12913-023-09767-z.

## Introduction

The Special Program for Research and Training in Tropical Disease (TDR) Global was established in 1974 and created a dynamic alumni community with the vision to improve “the health and well-being of people burdened by infectious diseases due to poverty through research and innovation”. Its mission is “to support effective and innovative global health research, through strengthening the research capacity of disease-affected countries and promoting translation of evidence into interventions that reduce burden of infectious diseases in the most vulnerable populations” [1, 2].

TDR has enduring investment in transfers of skills critical to sustaining resilient health research systems through the Postgraduate Training Scheme (PGTS), Clinical Research and Development Fellowship (CRDF), and Structured Operational Research Training Initiative (SORT IT) [3, 4]. By awarding research grants, sponsoring training in different laboratory skills and knowledge, building capacity of research facilities, and providing mentorship to researchers, TDR has contributed to efforts combating diseases of poverty in Ethiopia [5–7].

The Tropical Disease Research-Armauer Hansen Research Institute (TDR-AHRI) collaboration has undergone several stages of development with the early research work on leprosy by Drs. T. Godal and M. Harboe at AHRI [8, 9]. These professionals were later recognized globally for their influence on leprosy research and management. T. Godal, who served as the director of AHRI from 1970 to 73, later became the second director of WHO-TDR (1986–1998) an ascension likely facilitated by seminal work at AHRI. This has contributed to the continued synergetic relationship between AHRI and TDR [8]. Dr. Tore Godal was awarded The Prince Mahidol Prize in Public Health in 1999, and he donated interest on the monetary prize to AHRI to establish the Tore Godal Prize to recognize promising young Ethiopian scientists working on public health-related research projects. This prize is a testament to his commitment to Ethiopia and AHRI, and the highly competitive award is still given annually to deserving young Ethiopian researchers [9].

In the same spirit of commitment to keep AHRI and Ethiopian research irrepressible, TDR sent one of its experts, Dr. Howard Engers for a four-day visit to AHRI in January 2001 to review AHRI’s status on biomedical research in tropical diseases and discuss with AHRI scientists and partners on TDR’s call for proposal on operational research and/or clinical intervention research related to improving Tuberculosis (TB) control in Ethiopia [10–12]. This modest beginning led to the establishment of a successful, productive, and sustainable Tuberculosis Research Advisory Committee (TRAC) for Ethiopia [13]. The partnership has further contributed to the establishment of Pan-Africa Bioethics Initiatives (PABIN) and led significant changes in research ethics capacity in Ethiopia by large in the region [12].

Institutionally, TDR’s vision to “improve the health and well-being of people burdened by infectious diseases of poverty through research and innovation” complements AHRI’s vision “to become a leading biomedical research institute in generating scientific evidence for infectious diseases prevention and control” [1]. The long term complementarity and synergism of the two institutions is arguably understated and in need of documentation. Therefore, the purpose of this study was to explore the impact and lessons learned of TDR initiatives in Ethiopia in terms of capacity building, outcomes and implications.

## Methods

### Study setting

The study participants were recruited based on their engagement with TDR and or AHRI activities from Jimma, Addis Ababa, Gondar and Bahirdar universities, St. Paul Millennium Medical College, and staff members of selected international organizations. The engagement with TDR and/or AHRI was established through collaborative partnerships, including fellowships, grant awardees, and/or mentors for TDR programs and projects.

### Study design and period

A qualitative study with document review was conducted from May to July 2022. The study began with identification of the impact of the TDR’s support in Ethiopia by defining the inputs provided, the sustainment of efforts made at different levels and consequent outcomes in terms of expansion of research activities and the study was guided by the TDR Impact Pathways. The pathway outlined collaboration in research, conducting in-depth evidence synthesis, and utilizing the resulting insights to inform policy decisions and promote their implementation at a high level [2].

### Study participants

Twenty-three study participants from Jimma, Addis Ababa, Gondar and Bahirdar universities, St. Paul Millennium Medical College, and staff members of selected international organizations were involved in the study. Thirteen in-depth interviews, and five key informant interviews were completed. In this study, we identified individuals and institutes in Ethiopia that have collaborated with TDR in the roles of grantees, fellows, or mentors since the inception of the TDR-AHRI partnership. Our approach involved reaching out to these individuals to gain a comprehensive understanding of the extent and nature of their engagement and partnership with TDR.

### Data collection tool and procedures

Available reports, publications and written communications were reviewed with a focus on impacts and lessons learned related to policy and strategies, capacity and sustainability of health research, and bioethics in Ethiopia. During the review process, books, scientific publications, meeting minutes, and reports were utilized. Thirteen individuals who benefited from TDR support as trainees/fellows/grantees provided information on their individual experiences and five key informants from Gondar, Jimma, Addis Ababa Universities and AHRI provided information on institutional experiences.

Interview guides were developed to explore information on the impact and lessons learned of TDR initiatives in Ethiopia in terms of capacities built, outcomes and implications. The guides were prepared in English and translated to the Amharic language for easy communication (Supplementary file 1).

### Data management and analysis

Documents with a focus on TDR research support, AHRI-TDR collaborations and documents from service, academic, and research institutions that have had links to TDR and/or AHRI were extracted using a checklist. The checklist was developed with entries on geographical setting, type of research/report/document, time, to whom it addresses (public, academics, community, institutions), methods used, lessons, best practices, impact on research capacity strengthening, institution building, and gaps/challenges.

All interviews were audio recorded, transcribed verbatim, inductively coded and analyzed thematically as detailed below (Table 1). Once the transcription was completed, the researchers verified the text through parallel audio assessment., and all the transcripts were anonymized. The initial coding was made by KS and DFB while consistency was checked by MK and YK. Inter-coder differences were clarified, and consensus was reached among the researchers on the codes. The results were presented as themes with supportive verbatim quotes. N-Vivo software was used for data reduction and analysis. The study was reported following the Standards for Reporting Qualitative Research (SRQR) guideline.

**Table 1:** Lists of categories and themes

Theme	Sub-themes
1. Impact of TDR initiatives in Ethiopia	• Individual, • Institutional • National and Regional • Sustainability of research initiatives
2. Challenges to implement TDR initiatives	• Grant size • Tracking progress • Research dynamics and expectations • Institutionalization of collaboration • Administrative hurdles • Security concerns
3. Areas of improvement	• Grant size • Mentorship • Tracking progress • Institutionalization of collaboration • Administrative hurdles

### Trustworthiness

The interviews were led by the researchers themselves who were familiar with this research focus. To create a comfortable environment for discussion, interviews were conducted in settings preferred by the study participants. Data were read and re-read and coded by two of the researchers while two other research team members checked the consistency of codes. Data were triangulated by method, place and the individual participant’s profile. Efforts were made to maintain the original meanings when interpreting the data.

## Findings

### Study participant’s profile

As depicted in Table 2, out of the twenty-nine originally planned interviews, twenty-three participated and provided their responses. One-third of the participants were full professors, and five of these had a Ph.D. Over half of the participants were from academic institutions. Ten of the participants were grantees, seven were fellows and six were mentors.

**Table 2:** Study participant’s profile

Participants Identifier	Level	Educational level				Total
		PhD		Clinician/MSc		
		Male	Female	Male	Female	
Academic rank	Full professor	5	-	2	1	8
	Associate/Assistant prof	12	2	-	1	15
	**Total**	**17**	**2**	**2**	**2**	**23**
Institutional affiliation	Academia	10	-	1	1	12
	Research or MoH	3	2	1	1	7
	Non-governmental institution or WHO	4	-	-	-	4
	**Total**	**17**	**2**	**2**	**2**	**23**
Association with TDR	Grantee	8	1			9
	Clinical Research Development Fellowship (CRDF) or short-term fellow	7	1			8
	Mentor or consultant	6	0			6
	**Total**	**21**	**2**			**23**

### Impact of TDR initiatives in Ethiopia

#### Individual level empowerment

Findings from this study showed that TDR initiatives helped in enhancing human capacities. TDR provided a number of capacity-building training including fellowships, short-term training from a few days to six months, and support for MSc and Ph.D. programs. Areas of the training encompassed research ethics, good clinical practice (GCP), good laboratory practice (GLP), immunology, vaccinology and biotechnology, manuscript writing, quality management, and clinical trials.

*“In 2007, I took an immunology vaccinology course by TDR. The course was good. It was very inclusive with theory and laboratory. Fortunately, I got the training immediately one or two years after my MSc study that paved the way for my Ph.D. transition.’’ (Fellow, Addis Ababa)*.

A number of junior researchers in Ethiopia have obtained capacity-building opportunities through a one-year fellowship with a one-year re-integration plan, training and short-term courses on clinical trials, immunology and vaccinology, and scientific write-up.

*“I thank TDR for offering the free trial research training, which has been instrumental in guiding my professional journey by then.” (Fellow, Addis Ababa)*.

Another participant has also pointed out that;

*“The training helped me to start and strengthen my connection with colleagues those under the department that have direct or indirect involvement in neglected tropical disease (NTD) research, and they were from different countries including Africa, Asia, and Latin America” (Fellow, Addis Ababa)*.

Trainees, grantees, and fellows have underscored that their grant-winning capacity has significantly improved and helped them to be competitive for other bigger funding schemes and to publish research outcomes. Through the capacity building gained from TDR a fellow became the sole evaluator for a diagnostic tool. As it was indicated that;

*“The training helped me to evaluate diagnostic tools and machines in Ethiopia called Truenate. It is difficult if you do not have experts that can closely monitor. It could have been difficult for them to monitor from Geneva and frequently travel to Ethiopia.” (Fellow, Addis Ababa)*.

TDR support through SORT-IT enabled individuals to develop their careers and benefited Ethiopia through developed guidelines. SORT-IT has one of its 55 global collaborating institutes in Ethiopia. Review of SORT IT documentation and AHRI’s annual report indicated that there were 23 publications for 2009 to 2022 in connection to SORT-IT which has been supported by TDR.

*“SORT-IT publications have influenced policies and strategies. Good examples from Ethiopia include the national guideline which served as WHO consensus criterion, Podoconiosis – NTD master plan, and WHO’s guideline on surgical intervention.” (Grantee and mentor, Bahirdar)*.

### Institutional level empowerment

WHO/TDR has made a huge contribution to Ethiopia’s research portfolio and AHRI played a significant role to facilitate TDR support in Ethiopia. A typical example of this included GCP training center in AHRI, the establishment of tropical and infectious disease control center (TIDC) in Jimma, the Pan African Bioethics Initiative (PABIN) secretariat based in AHRI, and a survey on Research Ethics function in Africa during COVID19 pandemics hosted by Ethiopia.

*“TDR supported PABIN from the inception and the secretariat is AHRI. The AHRI TDR contribution was immense to bioethics in local universities. The support helped us to have strong capacity to run bioethics.” (Mentor and grantee, Gondar)*.

TDR facilitated Good Clinical Practice (GCP) training at AHRI that enabled AHRI to be the national GCP training center in Ethiopia. The first clinical trial in Ethiopia was done by AHRI with capacity-building support from TDR.

*“Through TDR support, AHRI is the sole institution that rolls the GCP initiative. It offers training on clinical trial and GCP for interested institutions and researchers throughout the country.” (Grantee and mentor, Addis Ababa)*.

A TDR grantee has established a tropical and infectious disease control center (TIDC) at Jimma University. This center has contributed enormously to research output to date as more than 50 Ph.D. students, young and senior researchers are conducting research in the center. Furthermore, Jimma University laboratories have been strengthened through the center and this, in turn, is supporting the development of malaria diagnosis and treatment kits for hard-to-reach populations in the area.

*“My first grant has been from TDR. It was for my Ph.D. and I used the money to establish my research center to conduct the laboratory analysis of my Ph.D. work as I told you; currently the center serves many students and researchers in Jimma university…” (Grantee and mentor, Jimma).*

The establishment of a biorepository at the Jimma University research center has been initiated and partly accomplished by a TDR fellow as part of the TDR reintegration plan.

*“The first reintegration plan was providing training to junior staff on clinical research and diagnostic[s], the second plan was capacity building to establish bio-banking and we switched to biorepository in our setup….’’ (Fellow, Jimma)*.

### National and regional level empowerment

TDR has supported several projects in Ethiopia including malaria and anemia control programs that became part of the Integrated Management of Childhood Illness (IMCI) program, nationwide NTD mapping, diagnosis, and establishment of a tropical and infectious disease control center at Jimma University (TIDC), establishment of the TB Research Advisory Committee (TRAC) in Ethiopia, strengthening research ethics at national level, and gender balance on malaria elimination. One of the participants pointed out that:

*“The center for tropical and infectious disease helps to strengthen Ethiopian research capacities. The diagnostic algorithms, tools, and diagnostic kits generated from this center and laboratories were disseminated to the ministry and the ministry used it in nationwide scale up.” (Grantee and mentor, Jimma)*.

Another participant explained that;

*“TDR had a research interest aiming to strengthen epidemiologic research mainly on leishmaniosis, and onchocerciasis. We got basic epidemiologic information in Ethiopia on leishmaniosis through TDR support.” (Grantee and mentor, Addis Ababa).*

TDR provided capacity building for Ethiopian Research Ethics professionals and Institutional Review Board (IRB) members. The team trained through this initiative developed a training course for researchers, research ethics committees of Ethiopia and across Africa on the important ethical consideration in research, and accreditation of IRBs through PABIN. While other African countries may have similar initiatives, in Ethiopia the Bioethics initiative has benefitted from TDR support through PABIN.

*“We gave several trainings under PABIN. The first training was given to our national ethics review board (in Ethiopia), then to the Addis Ababa University institutional review board. Afterward, we tried to give the training to regional IRBs. We went beyond Ethiopia, and gave the training in Uganda for trainees from all East Africa nations including Kenya, Tanzania, and Uganda.” (Grantee and mentor, Gondar)*.

Study participants from AHRI, AAU/CHS, and Jimma University affirm that TDR has contributed to strengthening laboratory capacity through funding schemes and equipment support. This was one of the prominent means of support for NTD elimination in Ethiopia, and continued to address the global health threats and neglected tropical diseases. Tuberculosis, malaria, and antimicrobial resistance have been other areas of focus supported by TDR. A nationwide NTD mapping, diagnosis, treatment, and laboratory strengthening has been supported by TDR, and TDR initiated prominent support for activities ensuring sustained elimination of visceral leishmaniosis. TDR support has also benefited Ethiopia by strengthening its capacity to conduct clinical trials partly through building clinical trial and training centers for GCP and supporting Ethiopian postgraduate students and researchers.

*“…after the training and grant, together with my mentor we renovated our laboratories in different universities…”. (Fellow, Addis Ababa)*

*“TDR gave the grant to AHRI for leishmaniosis mapping in Ethiopia; it enables (us) to answer basic questions on diagnosis and treatment options.” (Grantee and mentor, Addis Ababa)*.

TDR has contributed to the Ethiopian biomedical science research working group through funding, training, and fellowship opportunities. In line with this, the nationwide TB research advisory committee (TRAC) has been established.

*“TRAC is an important platform beyond Ethiopia. It is now researchers’ networking opportunity to share experiences, address challenges together and contributing to national TB program.” (Mentor, international)*.

TDR supports guideline development focusing on community engagement in research, mentorship within the scope of serving low and middle income countries (LMICs), and integrated management of childhood illness (IMCI) which is in practical use to date at national, regional, and global levels.

“*In collaboration with TDR nodes of the US and Latin America, we are leading the initiative to develop a guideline on the institutionalization of mentorship for LMICs.” (Grantee/mentor, Addis Ababa)*.

*“My project had a specific objective which was global capacity building. My research outputs were converted to guidelines and training material. More than 100 countries implement IMCI and currently, we are developing the material assisting with emerging technology. Within a few months, it will be launched.” (Grantee and mentor, Addis Ababa)*.

### Promoting Research Equity

Study participants indicated that TDR support fairly incorporated gender balance and TDR has also direct gender-related calls including the recent grant on gender balance in malaria elimination at Jimma University. TDR initiatives encompass disadvantaged groups by focusing on neglected tropical diseases mainly in low and middle-income countries. One of the prominent examples are initiatives in bioethics and NTDs.

*“… Among the trainees in ethics, most of us were female. I am a physician working in the countryside. So the chance given to me means the chance given to women living in the rural area.” (Grantee and mentor, Gondar)*.

### Sustainability of TDR initiatives

TDR initiated and supported postgraduate training with different interactive modules on biomedical research combined with laboratory activities at AHRI. Thereafter, AHRI has been serving as a center for this postgraduate program and taking responsibility of the entire initiative and it has now 74 postgraduate students (59 Ph.D. and 15 MSc). Through such support some universities in Ethiopia have already built their own postgraduate program training capacity and handle the training by themselves.

*“TDR is one of the most important collaborators. AHRI supports and trains universities. Example, [for] Haromaya University, Ambo, Gondar, and the like, we gave continuous Ph.D. and MSc training and that enables them to have their department. Haromaya is one good example to have their department.” (Grantee and mentor, Addis Ababa)*.

Because of TDR grants, Ph.D. programs in infectious disease control are running in Haramaya and Jimma universities with several students enrolled to date. TDR’s individual and institutional support enabled individuals to enhance their grant-winning and networking capacity. This contributed to retaining experienced and skilled staff at local universities and research institutes.

*“I could say, currently the TIDC [has become] become self-sustained which serves as [a] research center for Ph.D. students who are from different universities to conduct their research…” (Grantee and mentor, Jimma)*.

Since its establishment, TRAC has successfully organized sixteen annual research conferences. The 2022 conference was conducted on World TB Day at Hawassa university during March 22–24, 2022 with the theme of “Invest to End TB, Save Lives”. During this conference, the issue of spread of TB across borders was deliberated by national TB program managers from Eritrea, South Sudan, Somaliland, and Kenya. It was agreed to promote TRAC as a regional network to strengthen TB control partnerships.

*“TRAC is now beyond the conference. It focuses on TB research across countries in the horn of Africa, [gives] gave training on field epidemiology, facilitates preparation of several operational research. The outcome of which are of interest to the ministry of health to scale up…” (Grantee and mentor, Addis Ababa)*.

### Challenges in implementing TDR initiatives

Senior researchers indicated that the scope of TDR grant schemes was limited as its mainly focused on selected tropical diseases, and only small or seed grants have been offered. Study participants have also explained that in the past, TDR funds were small but important catalyzers and entry points for scale-up. Though such small grants have brought significant changes, participants felt that TDR should redefine its grant size to expand its contribution to science and or contribute to the increasing funding base for research.

*“I suggest if TDR could increase its funding size or catalyze[s] the opportunity to access such funds. Currently, there are more funding opportunities but accessing such fund[s] is not that easy. TDR’s support to improve capacities to access such fund may improve [the] number of researches and outcome.” (Mentor and grantee, Addis Ababa).*

In this study, it was described that small grants from TDR can show case successful research to draw the attention of more research funding institutions. As a result, currently senior TDR alumni are working with a number of other organizations that can provide considerably bigger funding.

*“I can say (since 2006 GC), we did not have much collaboration with TDR since there are other funding potentials. However, the small grants from TDR have contributed to my research portfolio of today. As research interest is growing and more young researchers are coming forth, TDR’s small grant and capacity building is critical. I suggest TDR to keep track of research activities in Ethiopia.” (Grantee and fellow, Addis Ababa)*.

Although TDR appears to have maintained its traditional disease focus of poverty which remains to be Malaria, TB, human immunodeficiency virus (HIV), and NTD, it has shifted priorities in approach towards implementation research and moved away from translational lab research. While that is the case at TDR, participants argued that AHRI is not moving as rapidly towards implementation research. One of the participants emphasized that,

*“Now, TDR is focusing on implementation research while the basic science, laboratory-based research activities are not TDR’s priority. AHRI may have to catch up with such [a] strategic global move to translate science to solve problems while it could support universities and research institutions in the region to take over AHRI’s usual research focus.’’ (Mentor, international)*.

Despite TDR’s support in research capacity and research initiatives, considerable internal barriers were mentioned that affect the pace at which research activities progressed. Such challenges were found to relate to the lengthy procedures in drawing money for the planned research activities, lengthy IRB approval processes (at local universities and national), and delays in shipment of biomedical supplies and equipment.

### Areas for improvement

Public health challenges in Ethiopia evolve with time, yet the diseases of poverty such as malaria, TB, HIV and several NTDs are still major public health problems. Moreover, emerging and re-emerging infectious diseases remain a challenge for public health programs. In view of existing research capacity, potential at universities and research institutions and the shifting global disease priorities, it is crucial to reconsider research priorities and approaches. TDR and AHRI should use their established relationship to work towards strategizing research focus and approaches in Ethiopia in line with global research direction.

*“Sometimes research outcome from focused research by a researcher may not bring much change. Perhaps broadening research focus and alignment with disciplines as well as the research community may help translate the research outcome to solve practical problem. TDR and AHRI’s support in this is desirable.” (Grantee, Jimma)*.

It was also suggested that AHRI as a TDR collaborator should align its structure, scope and direction to the current focus on implementation research. It is also recommended that AHRI should strengthen its own internal administrative and ethical review procedures to facilitate research undertaking while it supports universities and research institutions to follow suite.

## Discussion

Globally, healthcare services improved significantly with the advent of new technologies. However, there is still access limitation mainly in LMICs; some potential challenges include urbanization, climate change, animal-human interface, and lack of evidence-based healthcare practice. Response to such challenges has been taken up by TDR [14–16]. This is well articulated in its mission statement as “to support effective and innovative global health research, through strengthening the research capacity of disease-affected countries and promoting the translation of evidence into interventions that reduce the burden of infectious diseases and build resilience in the most vulnerable populations [17]. The TDR initiatives advocate and guide joint efforts to accelerate the achievement of sustainable development goals (SDGs) targets with a special focus to end the epidemics of HIV, TB, malaria, hepatitis and NTDs [18]. Such efforts help to draw attention of other stakeholders, including AHRI that are responsible for generating evidence for decisions, and program designers and managers for service delivery.

TDR has partnered with individuals as fellows or grantees, groups through training and institutions that served as centers to build capacities. Findings from this particular study revealed that TDR’s support to the different entities helped to promote remarkable results and useful lessons. Support to individual fellows and grantees, operational research group training through SORT IT and institutional research capacity building for AHRI, Jimma and Addis Ababa universities that has largely remained disconnected was found to have far-reaching impact [19].

AHRI has benefitted from research capacity-building support and accessing small grants from TDR for some specific research activities. Despite lack of a formal arrangement, such as a memorandum of understanding or core funding support, TDR and AHRI have many shared priorities. Thus, the relationship between the two institutions, the influence and contribution of TDR to AHRI’s biomedical and health research excellence, and its relevance and accountability to attract more funding for research is obvious [20].

This study articulated useful impacts that were reported by recipients of individual as well as institutional support from TDR. Support in the form of training on research competencies, provision of competitive research grants and fellowships to individuals, and institutional capacity-building through establishment of new or renovation of existing laboratories was impactful. Individuals felt empowered to design and undertaking operation research but also in writing successful grants. Validation of diagnostic tools, development of a malaria case management guide from the field laboratory in Jimma, systematizing TB sample storage and digitalization usable for genomic studies to facilitate research in the field and validation of new TB diagnostic tool were contributions from empowered individuals [10, 14]. Moreover, numbers of peer-reviewed publications from TDR and AHRI’s support depicts the fruition of research capacity building. The implication is believed to be far-reaching as such evidence may lead to useful contributions in science, program, and policy.

Institutional empowerment by TDR’s contribution was found to thrive with its contribution in the rollout of capacities within the country and beyond. Empowered institutions such as Jimma University and AHRI offered laboratory capacity (space, guidance, and resources) to facilitate research. This has enabled them to train health researchers at a different level of competence which could not have happened without the shared goal of the institutions involved. Several students at MSc and Ph.D. levels were trained in those laboratories. Promotion of such local institutions by TDR has enabled the former to contribute to human capacity building while in parallel expanding opportunities for others. This was evidenced by the active collaboration of AHRI with Haromaya University while similar collaboration is planned [20]. Such expansion of research competencies and capacities to local universities closer to the community does not only ensure sustainability of research capacity building initiatives but also brings research closer to the community.

Based on our research, participants highlighted the absence of continuity of initiatives that limits sustainability. Typically, support ends with the individual commitment to the project. One potential solution to this challenge is to promote equitable partnerships that can strengthen south-south collaborations. In the context of global health training and capacity building, a key consideration is to ensure the local power system is not influenced by western-driven agendas that do not take into account the contextual needs of the community. Thus, decolonizing the health system is a necessary step towards promoting equitable global health capacity building.

It was clear that lack of organized documentation of TDR initiatives and collaboration with institutions and individuals in those institutions impeded clarification of TDR’s impact. Although we report here the experiences of diverse participants, we have only interviewed a small sample of those in the country who have collaborated with TDR, therefore, further exploration in a broader national context is needed. It is also difficult to assess the unique contribution of TDR as activities are often impacted by several funding agencies.

## Conclusion

Useful lessons were drawn from TDR’s research capacity building in Ethiopia and its relationship with AHRI. AHRI, as the only biomedical and clinical research center of excellence in Ethiopia has followed suite in building the capacity of local academic and research institutions with expansion of research institutionalization and mentorship culture. The capacities created at different local levels have influenced health care procedures with introduction, testing, and branding of diagnostic tools and algorithms. The malaria case management guide and tools, integrated management of childhood illness guidelines, the establishment of biorepositories, validation of a new TB diagnostic tool, the establishment and increased regional scope of TRAC and facilitated ethical review and clearance processes were lesson learning initiatives impacted by TDR support as well as the active role of AHRI. Aside from contributions to science, programs, and policies the influence on professional development of individuals and visibility of the institutions has been substantial.

Although AHRI does not have formalized framework guiding its collaboration with WHO/TDR, the complementarity of research activities on the ground has allowed AHRI and WHO/TDR to align. That alignment is founded in the trust that the continued success of researchers initially supported and trained at AHRI can promote and increased capacity at the eventual institutions of such researchers. Interestingly, WHO/TDR aspires to improve health and well-being of people burdened by infectious diseases of poverty, AHRI aspires to be a leading research and training institute for infectious diseases of poverty. Accomplishments, impacts, and lessons from research interventions that were supported by WHO/TDR were shared between the two institutions without a formalized collaboration between the two institutions.

An important lesson and implication of this collaboration is that small innovations with limited seed contribution can improve capacity to attract more resources for research and novelties with seminal knowledge creation, and improved tools and approaches to advance solutions to health problems. Moreover, it has enabled individuals value the importance of continued guidance and mentorship within institutions during periods of limited support.

## Electronic supplementary material

Below is the link to the electronic supplementary material.

Supplementary Material 1

## Data Availability

All relevant data are within the manuscript. We provided the interview guides as a supplementary file (Supplementary file 1) while the raw data will be available upon reasonable request from the corresponding author.

## Abbreviations

AAU: Addis Ababa University
AHRI: Armauer Hansen Research Institute
CRDF: Clinical research and development fellowship
HIV: Human immunodeficiency virus
IRB: Institutional Review Board
NTD: Neglected tropical disease
PGTS: Port graduate training scheme
SORT it: Structured operation research training initiative
TB: Tuberculosis
TRAC: Tuberculosis research advisory committee
