# Supplementary material for: Contribution of the World Health Organization/ the special program for research and training in tropical disease (WHO/TDR’s) in institution building: lessons from Ethiopia
Source: BMC Health Serv Res. 2023 Jul 18;23:764. doi: 10.1186/s12913-023-09767-z (PMC10353139; doi:10.1186/s12913-023-09767-z)
Supplement: Supplementary file 1 — Supplementary Material 1 [file 12913_2023_9767_MOESM1_ESM.docx]

**KII/IDI Interview guide**

Date of interview: ________ Time interview started: _________Time interview ended: _______

Place interview conducted: ____________________Recording file name: _________________

| **1** | **Introduction** |
| --- | --- |
|  | Could you briefly you tell us about yourself and your experiences?  **Probe:** Age in years, Sex, Profession, Years of experience, Current position, Affiliation |
| **2** | **Key questions and probes**   1. Would you tell us about your experiences in TDR funded projects?   **Probe:** role in the project, number of current/previous grants, contribution on career level, advancement in research activities, financial support   1. Could you explain about the changes at personal level due to TDR projects??   **Probe:** Communication skill, writing skill, partnership opportunities, publications/promotions   1. How the TDR projects impact the community at large?   **Probe**: burden of communicable diseases, socioeconomic status, education, sanitation, health system, community-led approaches, community health workers, campaigns   1. How do TDR supports help your research capacity?   **Probe**: trainings, online courses, implementation research, grants, leadership in research, mentorship, contribution in creating resilient health research system   1. Could you briefly tell us the change brought due to the TDR projects?   **Probe**: Impact on the function of institution, policy and strategy input   1. How do you see TDR support in relation to ethical review process and regulatory procedures?   **Probe:** Experience with mentors, what were the gains   1. How do you see TDR support in relation to equity?   **Probe**: equal support to males and females, focus on disadvantageous group,   1. What are the barriers in applying to TDR grants?   **Probe**: training issues, research capacity, language barriers, approach of TDR administrators, focus areas of the grants, application procedure   1. What are the main challenges in implementing TDR projects in your institution/study area?   **Probe**: organizational bureaucracy, gaps in expertise capacity, sociocultural issues, infrastructure, finance, what measures taken to tackle the challenges   1. How can the challenges be improved?   **Probe**: partnerships and collaboration, networking, tools, and strategies   1. How can TDR projects impact the future health and wellbeing of people?   **Probe**: sustainability, Support the resilient health research system, mentorship |
| 3 | **Wrap up** |
|  | 9. Is there any other information about the impact of TDR support on research capacity strengthening?  Anything to say? |
